# Different healthy habits between northern and southern Spanish school children

**DOI:** 10.1007/s10389-017-0823-2

**Published:** 2017-08-14

**Authors:** Daniel Arriscado, Emily Knox, Mikel Zabala, Félix Zurita-Ortega, Jose Maria Dalmau, Jose Joaquin Muros

**Affiliations:** 1 Department of Education, International University of La Rioja, Logroño, La Rioja Spain; 2 School of Health Sciences, University of Nottingham, Nottingham, UK; 3 Department of Physical Education, University of Granada, Granada, Spain; 4 Department of Didactics of Musical, Plastic and Corporal Expression, University of Granada, Granada, Spain; 5 Department of Educational Sciences, University of La Rioja, Logroño, La Rioja Spain; 6 Department of Nutrition and Food Science, School of Pharmacy, University of Granada, Campus Universitario de la Cartuja s/n, P.C.:18071 Granada, Spain; 7 School of Medicine, University of Nottingham, Nottingham, UK

**Keywords:** Children, Healthy habits, Geographical location, Spain

## Abstract

**Aim:**

Healthy habits are influenced by several factors such as geographical location. The aims of this study were to describe and compare healthy habits within two populations of sixth-grade primary school children (aged 11–12 years) from northern and southern Spain.

**Subjects and methods:**

A cross-sectional study using two representative samples of school children was conducted. Participants came from Logroño (*n* = 329) in the north and Granada (*n* = 284) in the south of Spain. Socio-demographic and anthropometric variables, adherence to the Mediterranean diet, aerobic fitness, and healthy lifestyles were recorded.

**Results:**

Boys reported a higher level of physical activity and aerobic fitness than girls (*p* = 0.000). Southern school children reported significantly higher adherence to the Mediterranean diet (♀: *p* = 0.041; ♂: *p* = 0.008), lower aerobic fitness (♀: *p* = 0.000; ♂: *p* = 0.042) and hours of nightly sleep (♀: *p* = 0.008, ♂: *p* = 0.007) than northern school children. Southern boys also reported lower levels of physical activity (*p* = 0.013). There were slight or moderate correlations among all habits measured (physical activity, diet, screen and sleep time). Additionally, the physical activity level was inversely related to weight status. Overweight and obese northern boys reported less physical activity than healthy-weight northern boys (*p* = 0.020) and overweight and obese southern girls reported less physical activity than healthy-weight southern girls (*p* = 0.024).

**Conclusions:**

Results showed differences in physical activity, eating and sleep habits, and aerobic fitness according to geographical location. The relationships found among lifestyle habits indicate the need for health promotion interventions nationally and considering the differences discussed here.

## Introduction

Many risk factors for mortality and noncommunicable disease, such as high blood pressure and high blood glucose, are related to individual behaviours and lifestyle habits such as low consumption of fruits and vegetables and physical inactivity (Ezzati and Riboli 2013). These factors are also responsible for the overweight and obesity epidemic that affects more than 2 billion people across the world in both developed and developing countries (Ng et al. 2014). In Spain, more than a half of its population is overweight or obese (Ministry of Health, Social Services and Equality 2012).

Since these habits become moderate to highly stable over time (Pearson et al. 2011), it is important that healthy lifestyles are practiced from the early stages of life. Childhood is a key stage for the acquisition of behaviours likely to endure into adulthood and even for the rest of the lifespan. Engaging in physical activity and eating healthily are the most important habits that children should acquire to become healthy youths and adults (An 2015).

Healthy eating habits have a demonstrated positive influence on cardiometabolic (Funtikova et al. 2015) and mental (O’Neil et al. 2014) health in children and adolescents. Due to its geographical location, the traditional diet of the Spanish population is Mediterranean, rich in vegetables, fruit, legumes, nuts and cereals and with olive oil as the staple dietary fat. Unfortunately, current trends suggest that Spanish children are beginning to abandon the Mediterranean diet (MD) (García et al. 2015) despite its positive impact on health. Children and adolescents who defy this trend and report high adherence to the MD have a healthier cardiovascular profile and lower blood pressure (Farajian and Zampelas 2014), higher levels of health-related quality of life (Costarelli et al. 2013) and better academic performance (Esteban-Cornejo et al. 2016).

In the case of physical activity (PA), previous research has associated it with physical, psychological, social and cognitive health indicators in youth, especially when PA is performed at a moderate or vigorous intensity (Poitras et al. 2016). PA intensity is related to physical fitness, which is more strongly associated with cardiovascular risk factors than objectively measured physical activity alone (Hurtig-Wennlöf et al. 2007).

Other habits of children and adolescents related to health include sedentary behaviour and sleep time. Sedentary behaviour is often measured as screen time and is related to both higher adiposity and anxiety and low levels of fitness, quality of life, self-esteem and academic achievement (Saunders and Vallance 2017). On the other hand, short sleep duration, poor sleep quality and late bedtimes are associated with poor diet quality and obesity (Chaput and Dutil 2016).

Engagement in these habits is also influenced by various factors. These can be sociodemographic (e.g. age, gender, ethnicity, socioeconomic level), environmental (food availability, cost) or individual (eating rate, preferences) (Obregón et al. 2015). Further factors associated with PA and sedentary behaviours include active travel, school meal eligibility, lunch break duration and facilities (Morgan et al. 2016). It is important to identify the primary factors influencing diet and PA within specific populations to develop effective interventions.

Spain is divided into 17 regions, each with its own policies, lifestyles, economy and so on. The geographical location of the regions also creates climate differences especially between the south and the north. The aim of this study was to describe and compare self-reported adherence to healthy habits (diet, physical activity, sleep time, etc.) in two populations of sixth-grade primary school children (11–12 years) from northern and southern Spain.

## Materials and methods

### Subjects

The study used a cross-sectional design with two samples of sixth-grade primary school children aged between 11 and 12 years old, both representative of their regions. One sample came from Logroño (Rioja) in the north of Spain and one came from Granada (Andalusia) in the south of Spain. To be eligible for inclusion, children had to be without any major physical or behavioural disorder that could seriously impact participation. Research was conducted between 2013 and 2015. There are 1595 children attending 31 state and state-subsidised private schools in Logroño from which 372 were randomly selected and invited, and 337 agreed to participate in this study. The same sampling strategy was not employed in Granada as there are many more schools (*N* = 82) and it was not possible to make contact with all of them. Further, the sampling strategies for both Logroño and Granada were not initially developed for this comparative analysis but the interesting outcomes justified an exploratory comparative analysis between the two. Instead, for the Granada sample, 6 schools were selected from the 82 using cluster randomisation. All sixth-grade children of the six participating primary schools were then invited to take part in this study. This amounted to 306 children being invited to take part (5021 children attend six-grade primary school in Granada). Of these, 290 agreed to participate. Both children and their parents or guardians were informed of the objectives and methods of the study and told that they could withdraw at any time. Participants were instructed on how to fill out the questionnaires and how to conduct the tests. All tests were conducted during participants’ physical education lessons during school time. No incentives were provided to the children or parents. Fourteen children (eight from Logroño and six from Granada) were excluded for failing to complete some element of testing or because they failed to attend class on their testing day.

Ethical principles of the Declaration of Helsinki for medical research were adhered to. Informed consent was obtained from the parents or guardians of the school children. Ethical approval was granted by the Ethics Committee for Clinical Research of Rioja and the Ethics Committee of the University of Granada.

### Anthropometric measurements

Measurements were performed following the guidelines outlined by the International Society for the Advancement of Kinanthropometry (Stewart et al. 2011). Weight was calculated using an electronic scale (model 707, Seca Corporation, Columbia, MD; ±50 g accuracy). Height was determined using a stadiometer (GPM, Seritex, Inc., Carlstadt, NJ; ±1 mm accuracy). Body mass index (BMI) was calculated from height and weight. Overweight and obesity were defined according to international criteria (Cole et al. 2000).

### Level of adherence to the MD

The Mediterranean Diet Quality Index (KIDMED) questionnaire (Serra-Majem et al. 2004) was used to determine the level of adherence to the MD. This questionnaire consists of 16 items that relate to Mediterranean dietary patterns; items that denoted negative connotations with respect to the MD (e.g. do you eat candy daily?) were scored as a −1, while those with positive connotations (e.g. do you use olive oil for cooking at home?) were scored as a +1. The 16 items were then summed to produce a total score. Based on this score, participants’ adherence to the MD was classified as high (≥8), medium (4–7) or low (≤3). The school children were also asked whether they visited the school canteen on 3 or more days a week. All questionnaires were administered systematically by a trained researcher and supervised by three collaborators to ensure that all participants understood the questionnaires.

### Physical activity and maximal oxygen uptake

Physical activity levels were evaluated using the Physical Activity Questionnaire for Older Children (PAQ-C) (Kowalski et al. 2004). The questionnaire provides a general measure of physical activity for 8 to 20 year olds. The PAQ-C is a self-administrated questionnaire consisting of nine items rated on a five-point scale. A higher score indicates more active adolescents. Respondents are asked to recall the frequency and type of physical activity they have engaged in on each of the 7 days prior to completing the questionnaire. Validation studies have found the PAQ-C to be highly reliable (Saint-Maurice et al. 2014).

Maximal oxygen uptake (VO_2_max) was estimated using a 20-m incremental-maximum effort shuttle run field test (Léger et al. 1988). The test involves running to and fro between two lines placed 20 m apart. Participants start at an initial velocity of 8.5 km/h and increase their speed by 0.5 km/h/min until they can no longer reach the line on two consecutive occasions or when the participant can no longer maintain the physical effort required to continue. VO_2_max relative to body mass (ml/kg/min) was calculated using the established formula (Léger et al. 1988).

### Screen time, sleep time and physical activity habits

Participants were also asked the number of hours of sleep they achieved at night, their engagement in extracurricular school sports activities, daily screen time each day and how they travelled to school. They also reported their gender and date of birth.

### Statistical analysis

The means for all quantitative variables are presented alongside the standard deviation. Normality of the data was tested using the Kolmogorov-Smirnov test with Lilliefors correction and homoscedasticity was assessed using the Levene test. Quantitative values were compared using school children’s t-test for two-group comparisons for variables with normal distribution, with the rest analysed using the U Mann-Whitney. Qualitative variables are presented according to their frequency distribution and associations between them were determined using the chi-square test.

The association between quantitative variables was studied using the Pearson or Spearman correlation, depending on their distribution. Finally, a linear regression model was developed to identify determinants of the KIDMED score. Data were analysed using the IBM-SPSS version 21.0 statistical programme for Windows. The level of significance was set at 0.05.

## Results

Data for age, body mass index, aerobic fitness and lifestyle habits of the study participants according to gender are shown in Table 1. There were no significant differences according to gender in terms of age, body mass index or self-reported engagement in all lifestyle habits apart from physical activity. Boys reported higher levels of physical activity (3.19 ± 0.61 vs 2.89 ± 0.56). Aerobic capacity was also higher in boys than girls (45.70 ± 7.69 vs. 39.96 ± 7.63).

**Table 1:** Characteristics of study sample by sex

	Total (*n* = 613)	Girls (*n* = 321)	Boys (*n* = 292)	*P* value
Age (years)	11.88 ± .53	11.89 ± .52	11.87 ± .53	.659
Body mass index (kg/m^2^)	19.41 ± 3.44	19.23 ± 3.06	19.58 ± 3.73	.196
KIDMED	7.40 ± 1.93	7.43 ± 1.82	7.37 ± 2.05	.157
PAQ-C	3.03 ± .60	2.89 ± .56	3.19 ± .61	.000^**^
VO2max (ml/kg/min)	42.61 ± 8.58	39.96 ± 7.63	45.70 ± 7.69	.000^**^
Screen time (hours)	1.60 ± .92	1.59 ± .91	1.60 ± .93	.913
Hours of nightly sleep (hours)	9.39 ± 1.33	9.34 ± 1.31	9.44 ± 1.36	.397

*P < 0.05; **P < 0.01

Results showed that 26.8% of children were overweight or obese, without significant differences according to region or gender, although more cases of obesity were reported by boys. Similarly, there were no significant differences according to gender or region in relation to the likelihood of being categorised as having high adherence to the MD (49.3%), active transport to school (65.2%) or school canteen attendance (20.8%). However, differences did exist in terms of engagement in extracurricular school sports activities (66.2%), where girls registered less participation (*p* = 0.018).

Table 2 presents the differences between boys and girls from northern and southern Spain. Boys and girls were both significantly older in Granada as measurements were taken 2 months later than for the northern sample. Body mass index and screen time did not differ according to sex. Differences were identified for other measured variables. Northern girls reported more hours of nightly sleep (*p* = 0.008) and lower mean values for MD adherence (*p* = 0.041) and engagement in PA (*p* = 0.013). Regardless, they also showed greater aerobic fitness (*p* = 0.000). These trends were similar in northern boys, although self-reported PA was not significantly higher.

**Table 2:** Differences in body mass index, lifestyles and maximal oxygen uptake between northern and southern children

	Northern girls (*n* = 161)	Southern girls (*n* = 160)	*P* value	Northern boys (*n* = 168)	Southern boys (*n* = 124)	*P* value
Age (years)	11.75 ± 0.37	12.02 ± 0.61	0.000^**^	11.72 ± 0.37	12.06 ± 0.65	0.000^**^
Body mass index (kg/m^2^)	19.47 ± 2.73	18.97 ± 3.36	0.145	19.66 ± 3.42	19.49 ± 4.13	0.704
KIDMED	7.22 ± 1.74	7.64 ± 1.88	0.041^*^	7.10 ± 2.00	7.73 ± 2.07	0.008^**^
PAQ-C	2.81 ± 0.52	2.97 ± 0.59	0.013^*^	3.16 ± .55	3.24 ± 0.67	0.287
VO2max (ml/kg/min)	42.99 ± 3.60	35.75 ± 9.53	0.000^**^	46.57 ± 5.18	44.08 ± 10.75	0.042^*^
Screen time (hours)	1.55 ± 0.81	1.68 ± 1.09	0.328	1.60 ± 0.93	1.59 ± 0.94	0.896
Hours of nightly sleep (hours)	9.54 ± 0.66	9.15 ± 1.71	0.008^**^	9.64 ± 0.79	9.15 ± 1.85	0.007^**^

*P < 0.05; **P < 0.01

The influence of MD adherence, PA and aerobic capacity on body weight is presented in Table 3, according to gender and location. As shown in the table, an overweight or obese status was strongly associated with lower aerobic fitness in all groups. Additionally, healthy-weight northern boys (*p* = 0.20) and southern girls (*p* = 0.24) report higher levels of PA in comparison to overweight and obese northern boys and overweight and obese southern girls. Interestingly, no association was found between adherence to the MD and body weight in either population.

**Table 3:** Adherence to the MD, PA and aerobic fitness by sex and region according to body weight

			Healthy weight (*n* = 449)	Overweight and obese (*n* = 164)	*P* value
Northern	Boys (*n* = 168)	KIDMED	7.14 ± 2.03	6.98 ± 1.94	0.648
		PAQ-C	3.22 ± 0.54	2.99 ± 0.56	0.020^*^
		VO2max	47.90 ± 4.93	42.95 ± 4.03	0.000^**^
	Girls (*n* = 161)	KIDMED	7.22 ± 1.73	7.22 ± 1.78	0.997
		PAQ-C	2.78 ± 0.51	2.90 ± 0.56	0.174
		VO2max	43.49 ± 3.57	41.87 ± 3.44	0.008^**^
Southern	Boys (*n* = 124)	KIDMED	7.70 ± 2.08	7.71 ± 2.08	0.968
		PAQ-C	3.30 ± 0.65	3.09 ± 0.72	0.127
		VO2max	46.70 ± 10.65	37.68 ± 6.68	0.001^**^
	Girls (*n* = 160)	KIDMED	7.76 ± 1.78	7.64 ± 1.75	0.727
		PAQ-C	3.02 ± 0.56	2.77 ± 0.59	0.024^*^
		VO2max	37.66 ± 9.15	31.01 ± 9.45	0.003^**^

*P < 0.05; **P < 0.01

Table 4 shows the correlation coefficients among adherence to the MD, PA, screen and sleep time, and aerobic fitness. For northern children, greater adherence to the MD was associated with higher levels of physical activity (*r* = 0.22), lower levels of screen time (*r* = −0.34), more hours of nightly sleep (*r* = 0.13) and improved aerobic capacity (*r* = 0.15). Similarly, PA was correlated to lower levels of screen time (*r* = −0.17) and greater aerobic capacity (*r* = 0.45). Finally, screen time was inversely associated with nightly sleep (*r* = −0.21). For southern children, these patterns were similar in most cases; however, associations were weaker with some even losing significance. In fact, physical activity and screen time accounted for 13% of the variance in MD for northern children, but only 4% of variance for their peers in the south.

**Table 4:** Correlation coefficients between different lifestyles and aerobic capacity

Northern						Southern					
	MD	PAQ	Screen	Sleep	VO2max		MD	PAQ	Screen	Sleep	VO2max
MD	1	0.222^**^	−0.337^**^	0.131^*^	0.154^**^	MD	1	0.193^**^	−0.197^*^	0.047	0.014
PAQ	0.222^**^	1	−0.167^**^	−0.014	0.452^**^	PAQ	0.193^**^	1	−0.076	−0.140^*^	0.207^**^
Screen	−0.337^**^	−0.167^**^	1	−0.209^**^	−0.105	Screen	−0.197^*^	−0.076	1	−0.301^**^	−0.154
Sleep	0.131^*^	−0.014	−0.209^**^	1	0.044	Sleep	0.047	−0.140^*^	−0.301^**^	1	0.066
VO2max	0.154^**^	0.452^**^	−0.105	0.044	1	VO2max	0.014	0.207^**^	−0.154	0.066	1

*P < 0.05; **P < 0.01

MD, adherence to the Mediterranean Diet; Screen, screen time; Sleep, hours of nightly sleep; VO2max, maximal oxygen uptake

## Discussion

Of the present sample of school children, 26.8% were overweight or obese. This figure is slightly higher than reported in the last Spanish National Health Survey for 10 to 14 year olds (22.9%), although both samples found obesity to be more prevalent among boys (Ministry of Health, Social Services and Equality 2012). Weight status was not influenced by adherence to the MD in any case. The relationship between MD and obesity in children is not clear as the present findings contradict those previously reported (Farajian and Zampelas 2014). The different methodologies used by these studies could partly explain these differences, as could possible changes in physical activity or eating habits in between the first and second study being conducted. In the present study, 49.3% of school children demonstrated high adherence to the MD. This percentage is similar to that reported in the enKid (48.5%), a Spanish landmark study (Serra-Majem et al. 2004) and much higher than the overall percentage of the latest research conducted in children and adolescents using the same questionnaire (10%) (García et al. 2015).

PA engagement was inversely related to the BMI of both northern boys and southern girls. These results echo those found in other studies using objective methodologies, such as accelerometers, in which the PA level was negatively associated with risk of obesity (Hong et al. 2016). When questionnaires are used to measure PA, this relationship is not always evident (Laurson et al. 2008). Subjective tools could lack the power to identify subtle differences in behaviour. Further, it is more difficult to estimate the intensity of PA with self-report tools, but intensity could be an important component for obesity prevention in children (Laguna et al. 2013). In our study, boys reported engaging in significantly more PA than girls, as has also been reported by children and adolescents in other European countries (Van Hecke et al. 2016). Girls also reported lower participation in extracurricular school sports activities and had a lower maximal oxygen uptake than boys. Aerobic capacity was related to weight status in all groups. Previous research has identified body composition as the main predictor of maximal oxygen uptake in children aged 7 to 12 years old (Ara et al. 2010). The present research did not find any gender associations with body composition, eating habits, screen and sleep time.

The key findings from the present research concern the differences identified between northern and southern children. First, while southern children were not necessarily more likely to be in the high adherence category for MD, they did report higher mean values of MD adherence; this was the case for both boys and girls. In previous years, research has attempted to describe MD adherence in populations and other countries with equivocal results, while findings from one comparative review suggested that living in a Mediterranean country was not an important factor (Tognon et al. 2014). Another one found associations according to Mediterranean country (García et al. 2015). MD adherence in Spain has been previously reported. In 2004, the enKid study reported significantly higher adherence to the MD among children in southern regions (49.3%), including Granada, than among children in northern ones (37.5%), including Logroño (Serra-Majem et al. 2004). Another study that presented trends over the past 2 decades also shows adherence to be consistently higher in southern regions (Bach-Faig et al. 2011). In Italy, it also appears that adolescents from southern regions adhere to the MD more than those in northern regions (Noale et al. 2014). A number of factors could explain these findings. It is possible that the warmer climate and closer proximity to the Mediterranean Sea result in greater availability of Mediterranean products. However, other potential confounders such as genetic or environmental factors (agriculture, politics or socioeconomic status) should also be considered.

With regard to PA, southern school children also reported higher levels. Previous research examining physical activity engagement in various regions of Spain has produced inconsistent findings. For instance, enKid study data concluded that northern children and adolescents were more active than southern ones (Roman et al. 2008). On the other hand, a more recent report suggested slightly higher PA levels among southern adolescents (Moreno et al. 2016). These differences could be attributed to the different age ranges included in the studies or to a change in trends over the years. The present results also revealed higher aerobic fitness among northern children, especially girls. This is interesting given that northern children engaged in less PA. These findings could relate to the intensity of physical activity, which was not measured in the present study. Given the warmer climate in the south, children may be more likely to accumulate more minutes of outdoor activity and non-organised practise of a lower intensity. The cooler climate found in the north may result in children engaging in PA based on organised sport activities, which are typically performed at a higher intensity. Although findings were not significant (*p* = 0.09), northern children generally took part in more extracurricular school sports and activities. This kind of organised practise has been found to be associated with more minutes of moderate or vigorous PA, without necessarily increasing overall minutes of PA (Pearce et al. 2012). Additionally, a further study has concluded that sports participants demonstrate higher levels of fitness (Telford et al. 2016). It is possible that the PA of this northern sample is typically of a higher intensity than that of the southern sample, leading to their greater maximal oxygen uptake despite engaging in fewer minutes of PA. In line with previous findings (De Baere et al. 2016), the present results identified a relationship between cardiorespiratory fitness and PA, but with stronger associations in the northern children. This could also relate to the aforementioned implications of PA intensity.

With regard to sleep, southern children reported fewer sleep hours on week nights, with similar patterns for both boys and girls. The Health Behaviours School Children study reported the same trend with adolescents from Rioja sleeping slightly more hours than those from Andalusia (Moreno et al. 2016).

The present study identifies slight or moderate correlations among various lifestyle behaviours, often with stronger associations in the northern sample. Previous research has focused on factors that potentially influence eating habits. Higher adherence to the MD has been associated with a higher PA level and lower screen time in samples of Italian and Greek adolescents (Santomauro et al. 2014; Papadaki and Mavrikaki 2015). Adherence to the MD has also been associated with higher levels of fitness in a sample of Chilean children (Muros et al. 2017). The present research has corroborated previous findings pertaining to PA, screen time and MD, but has also elucidated relationships among other lifestyle behaviours. While hours of nightly sleep did not have an influence on body weight, it was related to other measured habits. In northern children, more hours of nightly sleep was directly associated with higher adherence to the MD and, in southern ones, inversely associated with the PA level. Additionally, it was inversely related to screen time in all children, suggesting that time in front of the television or other electronic devices could be displacing healthful nocturnal rest for some children. Research has previously warned that video device usage and the presence of a television in the child’s bedroom could negatively impact sleep duration and achievement of optimal sleep in children aged 1–14 years (Brambilla et al. 2017).

Two main recommendations emerge from the present findings. First, further research is necessary to determine the role of sleep time on child and adolescent behaviours and subsequent wellbeing. Further, it is evident that comprehensive health promotion is required throughout all regions of Spain and should integrate actions aimed at promoting physical activity, diet and sleep time and reducing sedentary time.

Limitations of the present study include its cross-sectional design, which precludes drawing conclusions on the direction of associations. Further, physical activity was measured using a validated questionnaire. Future studies should use accelerometry to provide more detailed and accurate information on physical activity intensity and sedentary time. The KIDMED questionnaire has inherent limitations of precision due to reliance on self-reported data and the choice of research method that precludes independent verification. However, the risk of error was minimised by ensuring the anonymity of responses. Further research is required to determine the generalisability of these findings across other geographical areas of Spain.

## Conclusions

The study results showed differences in physical activity, eating and sleep habits, as well as in aerobic fitness, according to geographical location, with southern children reporting higher levels of PA and adherence to the MD and less sleep time and aerobic fitness. The findings have important implications for the design of interventions for children and adolescents across Spain. Actions to promote improvements in PA, diet, screen and sleep time, which are comprehensive in nature and linked up in delivery, are likely to have the most positive impact on the intended recipients.
